# Resolution of tuberculosis blood RNA signatures fails to discriminate persistent sputum culture positivity after 8 weeks of anti-tuberculous treatment

**DOI:** 10.1183/13993003.00457-2024

**Published:** 2024-11-21

**Authors:** Claire J. Calderwood, Alvaro Sanchez Martinez, James Greenan-Barrett, Carolin T. Turner, Blanché Oguti, Jennifer K. Roe, Rishi Gupta, Adrian R. Martineau, Mahdad Noursadeghi

**Affiliations:** 1 Faculty of Infectious and Tropical Diseases, London School of Hygiene and Tropical Medicine, London, UK; 2 Division of Infection and Immunity, University College London, London, UK; 3 Department of Respiratory Medicine, University College London Hospitals NHS Foundation Trust, London, UK; 4 Institute of Health Informatics, University College London, London, UK; 5 Blizard Institute, Queen Mary University of London, London, UK; 6 C.J. Calderwood and A. Sanchez Martinez contributed equally; 7 A.R. Martineau and M. Noursadeghi are co-senior authors

## Abstract

**Background:**

Concerted efforts aim to reduce the burden of 6 months of anti-tuberculous treatment for tuberculosis (TB). Treatment cessation at 8 weeks is effective for most but incurs increased risk of disease relapse. We tested the hypothesis that blood RNA signatures or C-reactive protein (CRP) measurements discriminate 8-week sputum culture status, as a prerequisite for a biomarker to stratify risk of relapse following treatment cessation at this time-point.

**Methods:**

We identified blood RNA signatures of TB disease or cure by systematic review. We evaluated these signatures and CRP measurements in a pulmonary TB cohort, pre-treatment, at 2 and 8 weeks of treatment, and sustained cure after treatment completion. We tested biomarker discrimination of 8-week sputum culture status using area under the receiver operating characteristic curve (AUROC) analysis and, secondarily, assessed correlation of biomarker scores with time to culture positivity at 8 weeks of treatment.

**Results:**

12 blood RNA signatures were reproduced in the dataset from 44 individuals with sputum culture-positive pulmonary TB. These normalised over time from TB treatment initiation. 11 out of 44 cases with blood RNA, CRP and sputum culture results were sputum culture-positive at 8 weeks of treatment. None of the contemporary blood RNA signatures discriminated sputum culture status at this time-point or correlated with bacterial load. CRP achieved modest discrimination with AUROC 0.69 (95% CI 0.52–0.87).

**Conclusions:**

Selected TB blood RNA signatures and CRP do not provide biomarkers of microbiological clearance to support TB treatment cessation at 8 weeks. Resolution of blood transcriptional host responses in sputum culture-positive individuals suggests *Mycobacterium tuberculosis* may colonise the respiratory tract without triggering a detectable immune response.

## Shareable abstract

**Resolution of current blood RNA biomarkers of tuberculosis to normal levels is insufficient to stratify patients for treatment cessation at 8** **weeks, and illustrates uncoupling of host responses from persistence of viable bacteria in the respiratory tract** https://bit.ly/4bN2b1U

## Graphical abstract

**Figure:**
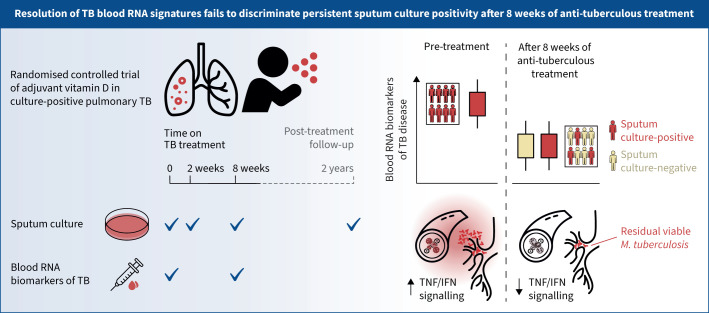
Overview of the study. TB: tuberculosis; TNF: tumour necrosis factor; IFN: interferon; *M. tuberculosis*: *Mycobacterium tuberculosis*.

## Introduction

Standard treatment for drug-sensitive pulmonary tuberculosis (TB) involves at least 6 months of combination anti-tuberculous treatment, which exceeds 95% cure rates in optimal settings [1]. Clinical trials of treatment regimens have reported >85% of people achieving cure with 3–4 months of treatment [2–6]. These underpinned a novel approach to reduce the treatment burden by using boosted 8-week anti-tuberculous regimens, followed by extended treatment for persistent disease, monitoring after treatment and retreatment for relapse. This approach achieved equivalent outcomes to standard care at 96 weeks with a 12% non-inferiority margin [7]. However, ∼20% of people experienced relapse during the follow-up period. Intensified post-treatment monitoring will be difficult to implement in stretched healthcare systems, and missed relapses likely to be more frequent outside of trial settings. Therefore, effective approaches to stratify individual-level risk of relapse at the end of shortened TB treatment regimens are required to enable implementation of these approaches in clinical practice. Persistent sputum *Mycobacterium tuberculosis* culture positivity would negate cessation of treatment. Therefore, we propose that a biomarker used to enable implementation of shortened treatment regimens should at least discriminate contemporary sputum culture status at the point of treatment cessation.

There has been sustained interest in tumour necrosis factor- and interferon-inducible blood RNA biomarkers of TB as triage tests for symptomatic TB [8–10] and for pre-symptomatic or incipient TB [11]. Taken together with the observation that their levels can change from the first week of therapy and normalise with treatment [12, 13], this piqued interest in their application to predict clinical outcomes or as a test of cure, with potential to provide greater sensitivity than resolution of symptoms and overcome the difficulties in obtaining sputum for microbiological testing. A number of studies have reported that these biomarkers discriminate contemporary microbiological treatment failure at the end of standard 6-month treatment regimens [12, 14–16]. Only one study has evaluated the performance of an 11-gene (Darboe11) signature to discriminate contemporary microbiological clearance before completion of 6 months of treatment, as a prerequisite for their potential utility to stratify risk of relapse following truncated treatment regimens. This showed statistically significant but modest discrimination of early and late sputum culture conversion after 8 weeks of treatment with a point estimate for area under the receiver operating characteristic curve (AUROC) of 0.73, but was limited to HIV co-infected individuals with recurrent TB [17]. It is not known whether this finding is generalisable to other gene signatures or to HIV-negative patients with their first episode of TB that may be candidates for shortened treatment regimens. We addressed this knowledge gap by testing the hypothesis that blood RNA biomarkers of TB discriminate early microbiological clearance (8-week sputum culture conversion) among people with pulmonary TB by undertaking a comparative analysis of candidate signatures, identified by systematic review, in a longitudinal clinical trial dataset [18]. We and others have shown that the long-established blood biomarker of inflammation, C-reactive protein (CRP), has comparable performance to blood RNA biomarkers of TB as a triage test for disease [19, 20]. Therefore, we also evaluated CRP as a test of microbiological cure/response to treatment at 8 weeks alongside our analysis of blood RNA biomarkers.

## Methods

### Study approvals

Blood sampling for transcriptomic analyses performed in the present study was approved by UK National Research Ethics services (reference number: 06/Q0605/83). All subjects provided written informed consent.

### Selection of biomarker signatures

We updated a previous systematic search (on 14 February 2023) to identify candidate blood RNA biomarkers, using the same databases and search strategy as described previously (supplementary methods and supplementary file S1) [8]. All records identified by the original and updated searches were sifted in duplicate (A. Sanchez Martinez, B. Oguti and C.J. Calderwood) to reflect updated inclusion criteria. In the current review, we included articles that described discovery of at least one concise whole-blood mRNA signature for the purpose of assessing the response to TB treatment, as well as those for diagnosis of active or incipient TB. Only signatures which had been validated in at least one separate dataset and were fully reproducible through a published calculation or regression equation were included. Signatures comprised of multiple genes are referred to as the surname of the first author of the corresponding publication, suffixed by the number of constituent genes.

### Study cohort and biomarker measurements

Genome-wide whole-blood transcriptomic data were obtained using cDNA microarrays, as previously described [21], in a subset of 46 people (supplementary table S1) with smear and culture-positive drug-sensitive pulmonary TB enrolled to a randomised controlled trial of adjunctive vitamin D therapy (AdjuVIT) [18]. We included a random sample from participants in whom sputum culture results were available at 8 weeks post-treatment, and longitudinal blood RNA samples were available pre-treatment and any of 2 weeks, 8 weeks or 2 years relapse-free survival post-treatment (supplementary figure S1). Sputum cultures and CRP measurements at 8 weeks post-treatment were performed as part of the study protocol in the original trial. Gram and Ziehl–Nielsen stains were used to confirm *M. tuberculosis* in positive MB/BacT liquid cultures. CRP levels after 8 weeks of treatment were previously reported, stratified by treatment arm and participant vitamin D receptor genotype [22], but not previously related to 8-week sputum culture status.

### Statistical power

The sample size was based on the number of cases from the AdjuVIT trial for which blood RNA, CRP and sputum culture results were available. We evaluated the power to discriminate sputum culture-positive and culture-negative cases from total sample size n=44 (for which contemporary biomarker and 8-week sputum culture results were available) stratified by ratio of positive/negative cases and AUROC, at 5% significance level (PASS2022 software; supplementary figure S2). With 25% of participants being culture-positive at 8 weeks, this study had 80% power to discriminate culture-positive and culture-negative cases with AUROC >0.75, at the 5% significance level.

### Data analysis

Analyses were performed using R version 3.6.1 (www.r-project.org). Blood RNA signature scores were calculated using the authors’ described methods. Blood RNA signatures with >20% of constituent genes missing, because they were not represented among protein-coding genes within the microarray dataset, were excluded (supplementary table S1). Signature scores were scaled by z-scores standardised to the mean and standard deviation of measurements from a subset of participants with sustained cure 2 years after treatment completion (n=31). The Wilcoxon rank-sum test was used to identify statistically significant differences between distributions of biomarker z-scores at different time-points. AUROC analysis was performed using the pRoc package to evaluate two-class discrimination and 95% confidence intervals with DeLong's method [23, 24]. Sensitivity and specificity were calculated at the maximum Youden index of the ROC curve, and prespecified thresholds of z-score of 2 (z2) for blood RNA signatures and 5 mg·L^−1^ for CRP, and their confidence intervals computed by the binconf function from the Hmisc package in R. The primary analysis focused on biomarker discrimination AUROC of sputum culture status at 8 weeks of treatment. In addition, we undertook secondary analyses to test the hypothesis that biomarker levels at 8 weeks correlated with quantitative bacterial load in sputum culture-positive individuals at 8 weeks of treatment using a linear regression model, and to evaluate discrimination of 8-week sputum culture status by biomarker measurements in the preceding time-points (pre-treatment and 2 weeks).

The inclusion criteria for selection of gene signatures in the present systematic review excluded the Darboe11 signature because this required rederivation by a support vector machine (SVM) learning model. However, we recently showed that the average expression of these genes performs as well as the original SVM-derived signature [20]. Since this signature had been previously reported to discriminate 8-week sputum culture conversion, we included the average signature score as a supplementary analysis in the present study.

### Data availability

The microarray data and associated metadata presented in this study are available at the European Bioinformatics Institute ArrayExpress repository with accession number E-MTAB-13605.

## Results

### Resolution of candidate blood RNA biomarkers of TB with curative treatment

1133 publications identified by systematic review yielded 38 studies and 49 blood RNA signatures of TB that met the criteria for inclusion. 37 out of the 49 blood RNA signatures were excluded from further analysis if they required rederivation of the model (n=16), they had lower accuracy than another signature from the same study (n=12), were not originally derived as a signature for TB prevalent or incident TB disease (n=3) or had >10% missing genes (n=6). 12 blood RNA signatures were included in downstream analyses (supplementary figure S3, supplementary table S2 and supplementary file S1).

**FIGURE 1:**
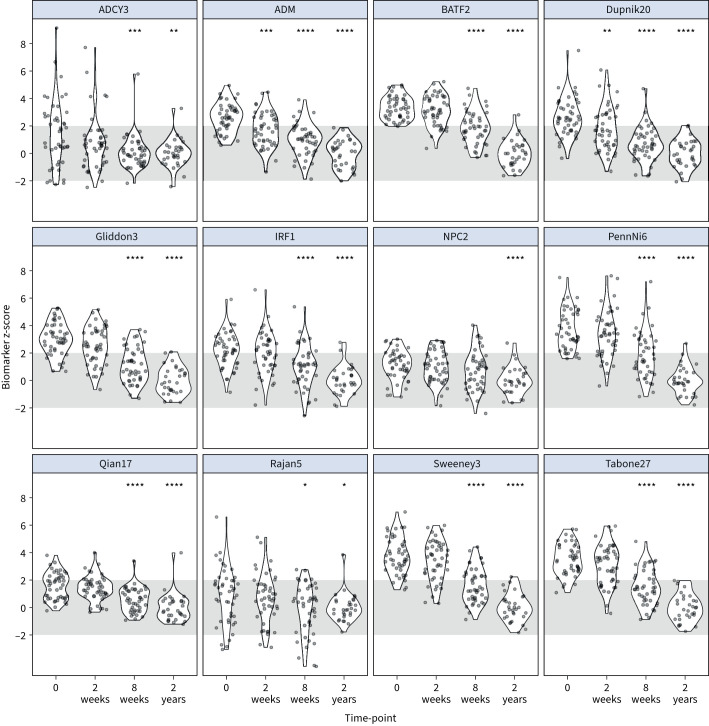
Longitudinal blood RNA signature scores by time from start of tuberculosis treatment. Blood RNA signature scores standardised to the distribution of 2-year post-treatment samples are shown pre-treatment (time-point 0), and 2 weeks, 8 weeks and 2 years from initiation of treatment. The 2-week, 8-week and 2-year time-points were compared to pre-treatment by the Wilcoxon rank-sum test. p-values for change from baseline to time-point indicated by: *: p<0.05; **: p<0.01; ***: p<0.001; ****: p<0.0001. Grey shaded area indicates z-score ±2 (normalised to 2-year values).

Blood RNA signature scores normalised over time from TB treatment initiation (figure 1). There was no significant difference in these signature scores between patients stratified by trial arm to receive vitamin D or placebo (supplementary figure S4). Consistent with their identification as biomarkers of active TB disease, most of these showed high classification accuracy for discriminating between pre-treatment measurements and 2-year post-treatment measurements (AUROC range 0.67–0.99) (supplementary figure S5 and supplementary table S3). At the prespecified z2 threshold, which approximates specificity to 98% by virtue of standardising biomarker scores using the distribution of 2-year post-treatment scores, the blood RNA signatures achieved sensitivity point estimates of 20–98% for identification of TB disease represented by the pre-treatment samples. A statistically significant reduction in the distribution of blood RNA signature scores compared to pre-treatment baseline measurements was evident at 2 weeks for one single gene signature (ADM), and for all other signatures by 8 weeks of treatment (figure 1).

### Biomarker discrimination of sputum culture status at 8 weeks post-treatment

**FIGURE 2:**
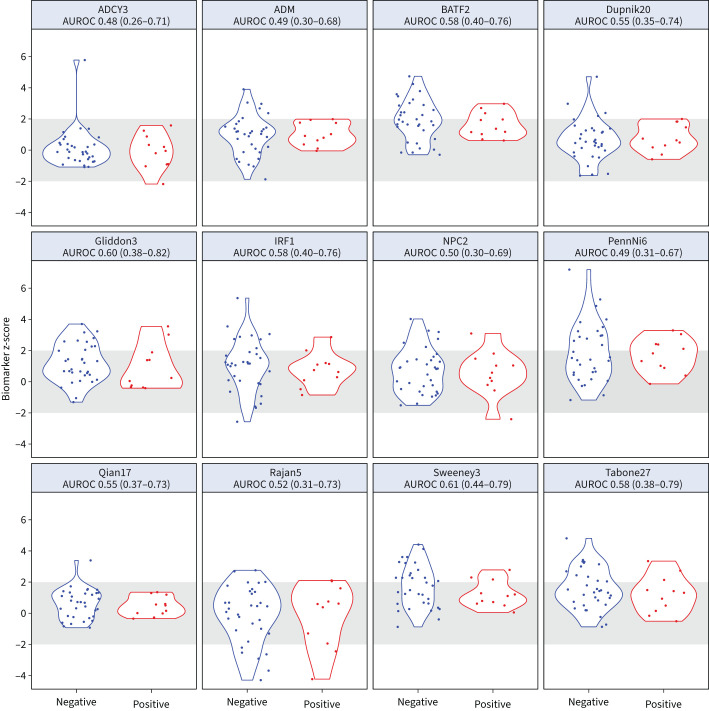
Blood RNA signature scores and sputum culture status at 8 weeks. Distributions of blood RNA signature scores at 8 weeks of treatment for tuberculosis, stratified by contemporary sputum culture status, showing area under the receiver operating characteristic curve (AUROC) and associated 95% confidence intervals for each signature. Grey shaded area indicates z-score ±2 (normalised to 2-year values).

11 out of 44 patients with blood RNA data available at 8 weeks of treatment had contemporary *M. tuberculosis* culture-positive sputum (supplementary figure S1). There was no evidence that any of the 12 blood RNA signature scores tested could discriminate between sputum culture-negative and culture-positive participants at this time-point, with AUROC point estimates of 0.48–0.61. CRP achieved the best AUROC of 0.69 (95% CI 0.52–0.87) (figure 2, supplementary figure S6 and supplementary table S4). Importantly, using the z2 threshold as the upper limit of normal range derived from patients with sustained cure 2 years post-treatment, we found most sputum culture-positive individuals at 8 weeks with blood RNA and CRP measurements in the normal range, indicative of the presence of viable bacteria without a discernible host response.

**FIGURE 3:**
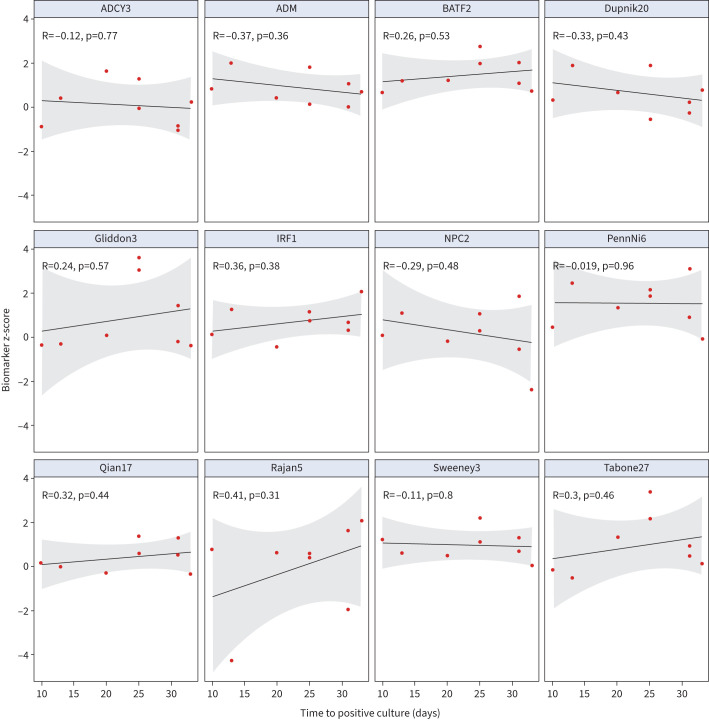
Relationship between blood RNA signature and bacterial load at 8 weeks. Scatter plots of blood RNA signature scores with sputum culture time (days) to positivity as a surrogate of bacterial load at 8 weeks of tuberculosis treatment showing individual data points and linear regression model (with 95% confidence intervals) for sputum culture-positive cases.

We performed three exploratory secondary analyses. First, we reasoned that the magnitude of the host response as reflected by the host response blood RNA signatures may be quantitatively related to bacterial load. We found nine out of 12 blood RNA signatures inversely correlated with time to culture positivity pre-treatment (supplementary figure S7), but this relationship was not evident for any of the signatures among patients who remained sputum culture-positive after 2 weeks (supplementary figure S8) or 8 weeks of treatment (figure 3). Second, we tested the hypotheses that the host response pre-treatment, early resolution of host responses at 2 weeks post-treatment initiation or the rate of resolution of host responses on treatment over the first 8 weeks may discriminate sputum culture status at 8 weeks (supplementary figures S9 and S10). No significant discrimination was evident by any of the biomarkers at baseline or by the change in biomarker levels between baseline and 8 weeks. Three out of 12 blood RNA signatures showed modest discrimination at 2 weeks with lower bounds of AUROC confidence intervals ranging from 0.53 to 0.55 (supplementary table S5). Third, we tested the performance of the Darboe11 signature that had previously been reported to provide some discrimination between sputum culture-positive and culture-negative cases after 8 weeks of treatment among HIV co-infected people with pulmonary TB [17]. Herein, we used the average expression of the genes in this signature which we found to be equivalent to the original SVM model as a biomarker of active TB [20]. The performance of the modified Darboe11 signature was comparable to all other signatures in our analysis. This signature normalised with time on treatment, but did not discriminate sputum culture status or relate to bacterial load at 8 weeks of treatment (supplementary figure S11).

## Discussion

To the best of our knowledge, we report the first evaluation of the resolution of blood RNA biomarkers of TB and CRP as a test of sputum culture conversion to reflect microbiological clearance following 8 weeks of anti-tuberculous treatment for TB. Even though elevated pre-treatment biomarker levels reduced to levels within the prespecified normal range by 8 weeks of treatment, none of the blood RNA biomarkers showed statistically significant discrimination of contemporary sputum culture status at this time-point. CRP achieved marginal albeit statistically significant discrimination. In addition, we found no consistent relationship between biomarker levels at earlier time-points or their response to treatment with 8-week sputum culture conversion. A correlation between host response biomarkers and sputum bacterial load in pulmonary TB has been reported in longitudinal analyses that include pre-treatment samples [25]. In our analysis, pre-treatment host response biomarkers were associated with bacterial load as continuous variables, but we found no relationship between contemporary bacterial load and biomarker levels stratified by time-point after 2–8 weeks of treatment. These results suggest that resolution of the current repertoire of blood RNA signatures of TB and CRP is unlikely to provide host response surrogates of contemporary microbiological clearance.

Our findings extend the evidence for the concept that the host response may be “decoupled” from the clearance of *M. tuberculosis* from the lung. Importantly, given blood RNA biomarkers can detect pre-symptomatic incipient TB [8], our data provide the most direct evidence to date for persistence of viable *M. tuberculosis* within the respiratory tract without a discernible disease-associated host response. This interpretation is further supported by our recent report of detection of *M. tuberculosis* DNA in circulating haematopoietic stem cells without perturbation of the blood transcriptome providing evidence for the existence of *bona fide* latent infection, within the limitations of current tools [26]. Compartmentalisation of selected host responses to *M. tuberculosis* infection have been reported previously [27]. Therefore, blood RNA signatures may reflect a host response to some *M. tuberculosis* subpopulations, rather than all *M. tuberculosis*, and these populations/compartments may have differential rates of clearance on starting treatment. Since sputum culture conversion only measures the bacilli that can be expectorated, these two tests may not be measuring the same *M. tuberculosis* populations. Testing blood biomarkers against culture conversion, as a proxy for disease activity, may therefore never achieve a test of microbiological cure. Importantly, sputum culture conversion at 8 weeks has not faithfully predicted long-term treatment outcomes in clinical trials [28]. Mycobacterial genetic material has been detected in pulmonary samples long after TB cure, suggesting that residual bacillary material is not exclusively associated with disease [29]. Therefore, it may be that blood RNA biomarkers at the time of treatment cessation using truncated regimens can predict long-term disease relapse even if they cannot predict contemporary microbial sterilisation.

Strengths of this study include the use of a comprehensive systematic review to select transcriptional signatures for head-to-head analysis alongside that of CRP. Quantitative bacterial load data from sputum cultures also enabled us to test for continuous relationships between host response and bacterial load on treatment. The small sample size of the study population is a limitation but does not detract from the observation that normalisation of blood RNA signatures at 8 weeks did not provide an adequate test of *M. tuberculosis* clearance from sputum at this time-point. Some of the reconstructed signatures were incomplete because they contained genes, microRNAs or pseudogenes that were missing in the AdjuVIT dataset. Nonetheless, they achieved comparable accuracies to the complete signatures. Some blood RNA signatures were excluded completely because we were not able to reconstruct them adequately in our dataset. However, previous analysis that shows co-correlation of these signatures [8] suggests that our findings are likely to be generalisable to other blood RNA signatures of active TB.

In view of the limitations of sputum culture conversion as an end-point, future evaluation of these signatures should be undertaken in treatment-shortening trials to assess their ability to discriminate patients with and without relapse after the end of treatment. Such studies should continue to incorporate genome-wide transcriptional profiling to support discovery of novel signatures for this application and head-to-head analysis of different signatures as they emerge.

## Supplementary material

10.1183/13993003.00457-2024.Supp1 **Please note:** supplementary material is not edited by the Editorial Office, and is uploaded as it has been supplied by the author.
Supplementary file 1 ERJ-00457-2024.File_1
Supplementary methods, tables and figures ERJ-00457-2024.Supplement

## Shareable PDF

10.1183/13993003.00457-2024.Shareable1 This PDF extract can be shared freely online.
Shareable PDF ERJ-00457-2024.Shareable

## Data Availability

The microarray data and associated metadata presented in this study are available at the European Bioinformatics Institute ArrayExpress repository with accession number E-MTAB-13605.
