# Supplementary material for: Resolution of tuberculosis blood RNA signatures fails to discriminate persistent sputum culture positivity after 8 weeks of anti-tuberculous treatment
Source: Eur Respir J. 2024 Nov 21;64(5):2400457. doi: 10.1183/13993003.00457-2024 (PMC11579542; doi:10.1183/13993003.00457-2024)
Supplement: Supplementary file 2 [file ERJ-00457-2024.Supplement.pdf]

Supplementary methods and data

Supplementary methods ... 2
Search strategy for blood RNA signatures ... 2
Eligibility criteria for candidate signatures ... 2
Data extraction ... 2
Missing gene symbols ... 2
Supplementary Table 1 ... 3
Summary characteristics of cases. ... 3
Supplementary Figure 1 ... 4
Consort diagram for samples included in the present analysis. ... 4
Supplementary Figure 2 ... 5
Statistical power of sample size ... 5
Supplementary Figure 3 ... 6
PRISMA diagram for selection of blood RNA signatures of TB by systematic review. ... 6
Supplementary Table 2 ... 7
Composition of blood RNA signatures included ... 7
Supplementary Figure 4 ... 8
Blood RNA signature scores stratified by time and AdjuVIT study arm ... 8
Supplementary Figure 5 ... 9
Blood RNA signature discrimination of pre-treatment and 2-year post treatment samples from AdjuVIT study ... 9
Supplementary Table 3 ... 10
Performance metrics of blood RNA signatures for discrimination of pre-treatment and 2-year post treatment samples from AdjuVIT study. ... 10
Supplementary Figure 6 ... 11
Biomarker discrimination of contemporary sputum culture result at 8 weeks TB treatment. ... 11
Supplementary Table 4 ... 12
Performance metrics of blood RNA signatures for discrimination of sputum culture positive and negative cases at 8 weeks TB treatment. ... 12
Supplementary Figure 7 ... 13
Correlation between pre-treatment blood RNA signature scores and sputum culture time to positivity ... 13
Supplementary Figure 8 ... 14
Correlation between blood RNA signature scores and sputum culture time to positivity at 2 weeks after treatment initiation. ... 14
Supplementary Figure 9 ... 15
Longitudinal blood RNA signature scores stratified by sputum culture status at 8 weeks TB treatment. ... 15
Supplementary Figure 10 ... 16
Longitudinal CRP measurements stratified by sputum culture status at 8 weeks TB treatment. ... 16
Supplementary Table 5 ... 17
Discrimination of sputum culture status at 8 weeks by blood RNA measurements at baseline, 2 weeks post treatment initiation and change in levels between baseline and 8 week post treatment initiation. ... 17
Supplementary Figure 11 ... 18
Performance of modified Darboe11 signature ... 18

Supplementary methods

Search strategy for blood RNA signatures

We searched Medline and Embase on 14/02/2023, restricting to articles published after the date of our previous search (15/04/2019), using the same search terms as previously. The search included comprehensive terms for 'biomarkers' (any of Biomarkers/Diagnostic Tests, Routine/"Predictive Value of Tests"/diagnostic test*.mp./biomarker*.mp./ppv.mp./npv.mp./sensitivit*.mp./specificit*.mp./signature*.mp.) AND 'tuberculosis (any of exp TUBERCULOSIS/tuberculosis.mp./exp MYCOBACTERIUM TUBERCULOSIS/tb.mp.) AND 'transcriptome' (any of RNA/Transcriptome//rna.mp./transcript*.mp./gene expression.mp./Gene Expression Profiling/RNA, Messenger/Transcription, Genetic/Gene Expression/) AND 'blood' (any of blood/lood.mp.), limited to human data. We consolidated the search by hand-searching reference lists of relevant review articles. New search results were merged with the results of the previous search and screened by two independent reviewers.

Eligibility criteria for candidate signatures

We included concise whole blood mRNA signatures discovered with a primary objective of either diagnosis of active or incipient TB, compared to controls who were either deemed healthy, or had latent TB infection, or, assessing the response to TB treatment, either as longitudinal change over time or with reference to another marker of treatment response (e.g. culture conversion, treatment failure or relapse). We defined 'concise' as signatures that used a defined approach to feature selection to reduce multidimensionality and the number of constituent genes, thus leading to biomarkers that may be more amenable to clinical translation, as previously. Other inclusion criteria were that the publications included validation of performance in cases/controls not included in discovery of the signature, availability of the gene names that comprise the signature, availability of the equation or modelling approach. We excluded gene signature models that required de novo derivation of the model in the original data set. Where genes required for a signature were missing from the analysis (AdjuVIT) dataset, we created a 'restricted' signature in the original dataset and compared the results to those obtained using the 'full' signature. If we were unable to reproduce statistically comparable results to those reported, the signature was excluded. Where multiple signatures were discovered for the same intended purpose and from the same training dataset within individual publications, we included the signature with greatest accuracy (as defined by the area under the receiver operating characteristic curve (AUROC) in the validation data), or if accuracy was equivalent, we included the most parsimonious signature.

Data extraction

Data were extracted using a pre-defined template by one reviewer and independently checked by a second.

Missing gene symbols

We reviewed gene symbols in g:PROFILER (https://biit.cs.ut.ee/gprofiler/convert) to identify all gene symbol and map to AGILENT microarray probe ID available in the analysis dataset. Signatures with greater than 20% genes missing were excluded from the downstream analysis.

Supplementary Table 1

Summary characteristics of cases.

		Included in analysis		Excluded from analysis		
		Sputum culture converted at 8 weeks		Sputum culture converted at 8 weeks		
**Characteristic**		**No**, N = 11[1]	**Yes**, N = 35[1]	**No**, N = 21[1]	**Yes**, N = 53[1]	**Not known**, N = 38[1]
Age (years)		36 (31, 42)	29 (24, 36)	34 (29, 40)	30 (25, 41)	37 (27, 53)
Gender	Female	1 (9.1%)	7 (20%)	4 (19%)	14 (26%)	14 (37%)
	Male	10 (91%)	28 (80%)	17 (81%)	39 (74%)	24 (63%)
BMI (kg/m^2)		20.21 (19.77, 21.12)	20.15 (18.48, 21.48)	18.93 (17.63, 20.42)	19.92 (17.96, 22.26)	19.09 (17.31, 21.88)
Days to positive culture[2]		11.0 (8.0, 13.5)	13.0 (9.0, 21.0)	9.0 (5.5, 11.0)	10.0 (8.0, 15.0)	11.0 (8.0, 13.4)
Ethnicity[3]	BLAF	2 (18%)	11 (31%)	5 (24%)	24 (45%)	9 (24%)
	EASIA	1 (9.1%)	3 (8.6%)	0 (0%)	3 (5.7%)	2 (5.3%
	EURAM	4 (36%)	3 (8.6%)	11 (52%)	7 (13%)	14 (37%)
	SASIA	4 (36%)	18 (51%)	5 (24%)	16 (30%)	13 (34%)
Educated >18 years		2 (18%)	20 (57%)	8 (38%)	26 (49%)	14 (37%)
CXR cavitation	Absent	5 (45%)	13 (37%)	6 (29%)	26 (49%)	23 (61%)
	Present	6 (55%)	22 (63%)	15 (71%)	27 (51%)	15 (39%)
Sputum smear grade[4]	≤1+	5 (45%)	23 (66%)	3 (14%)	25 (47%)	17 (52%)
	>2+	6 (55%)	12 (34%)	18 (86%)	28 (53%)	16 (48%)
CRP / mg/L[5]		42 (26, 82)	50 (28, 66)	73 (50, 100)	58 (38, 86)	56 (29, 76)

[1]Median (IQR) or Frequency (%). [2]Days to positive culture available on 43 people included in present analysis and 84 people not included. [3]In addition, two people of MIDEAST and one person of mixed ethnicity were in the cohort not included in the current analysis. [4] 5 people (all not included in the analysis) were missing baseline smear status. [5] 26 people (1 included in analysis and 25 not included) had missing CRP. BLAF= Black African, EASIA=East Asian, EURAM= European/American, SASIA=South Asian.

Supplementary Figure 1

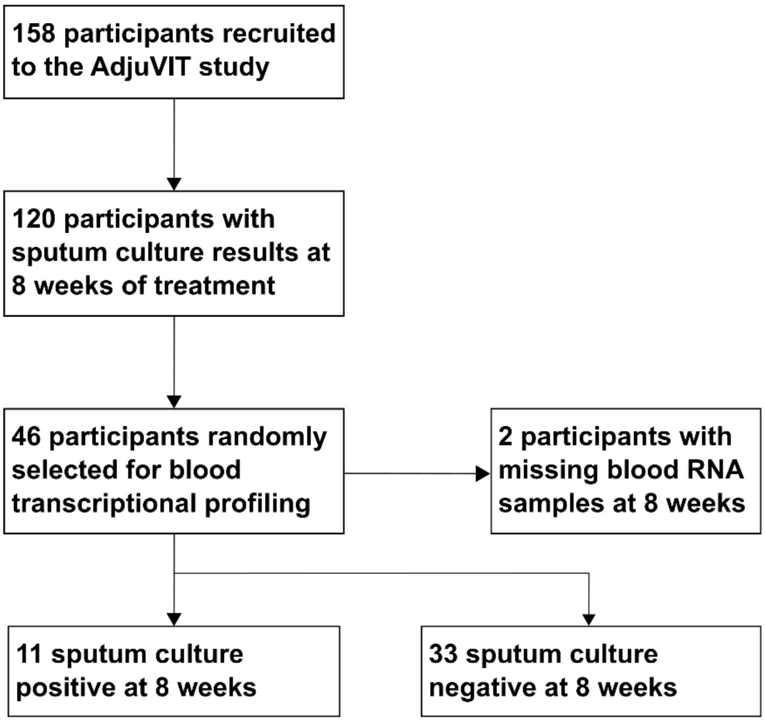

Consort diagram for samples included in the present analysis.

Supplementary Figure 2

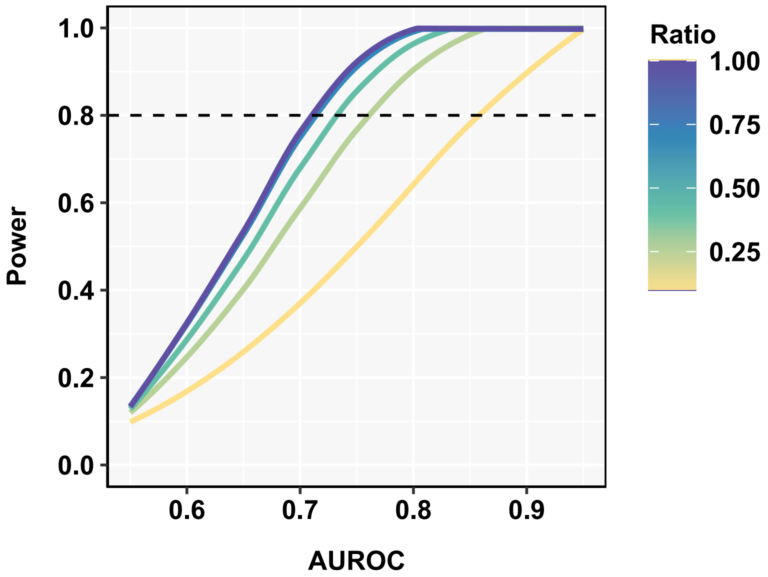

Statistical power of sample size

Power to identify statistically significant ($p<0.05$) discrimination of 8-week sputum culture positive and culture negative cases from total sample size N=44 stratified by ratio positive:negative cases and AUROC for which biomarker data were available at 8 weeks (PASS2022 software). These calculations used one-sided test to reflect the a priori hypothesis that biomarker measurements at 8 weeks will be lower in sputum culture negative cases compared to sputum culture positive cases.

Supplementary Figure 3

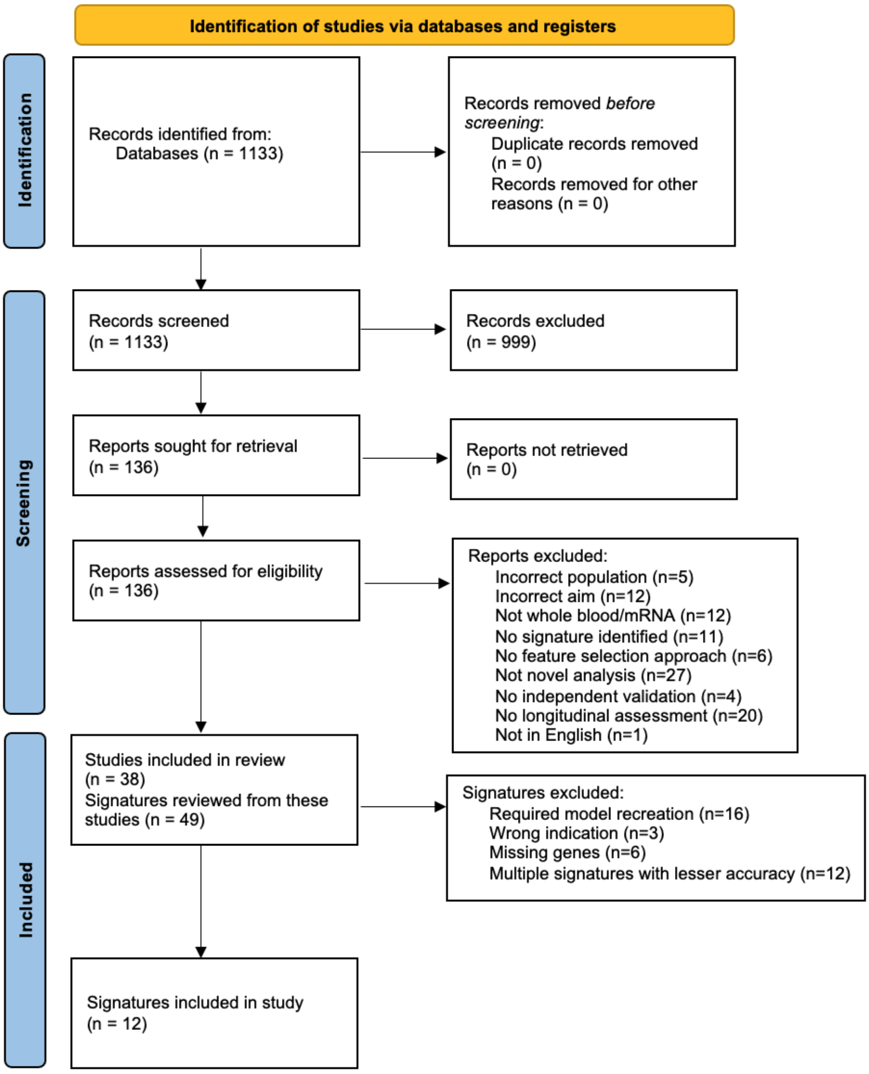

PRISMA diagram for selection of blood RNA signatures of TB by systematic review.

Supplementary Table 2

Composition of blood RNA signatures included

Blood RNA signature	Total genes	N missing	% missing	Score calculation
ADCY3	1	0	0	Single gene score
ADM	1	0	0	Single gene score
BATF2	1	0	0	Single gene score
Dupnik20	20	2	10	Sum(NAIP,ANXA3,SIPA1L2,CREB5,CCM2,SRPK1,SOCS3,PGLYRP1,LIMK2,CR1,CACNA1E,GYG1,DYSF,RAB20,PFKFB3,MAPK14,ENTPD1,PHTF1,WDFY3,GK)
Gliddon3	3	0	0	Sum(FCGR1B,C1QB,ZNF296)
IRF1	1	0	0	Single gene score
NPC2	1	0	0	Single gene score
PennNi6	6	0	0	mean(GBP2,FCGR1B,SERPING1)-mean(TUBGCP6,TRMT2A,SDR39U1)
Qian17	17	3	18	Sum(MT2A,HERC5,PSMB9,MOV10,PLAAT4,ISG20,WDFY1,GBP1,OAS1,PARP9,IFITM3,GCH1,SAMD9,STAT1,SCARB2,HLADMA,HLADMB)
Rajan5	5	0	0	Sum(GBP6,ACTA2,MTRF1L,GYG1,RABL2A)
Sweeney3	3	0	0	(GBP5+DUSP3)/2)-KLF2
Tabone27	27	2	7	Mean(CDCP1,C1QC,GBP6,C1QB,PRTN3,BAK1,ICAM1,SCARF1,IGF2BP3,SEPTIN4,SOCS3,LIMK1,GRAMD1C,GRIN3A,IL22,NRN1,CASP5,FCGR1BP,FCGR1CP,FCGR1A,GBP5,SDC3,PDCD1LG2,TNFSF11,P2RY14)
Darboe	11	1	9	Mean(BATF2,ETV7,FCGR1A,GBP1,GBP2,GBP5,SCARF1,SERPING1,STAT1,TAP1)

Supplementary Figure 4

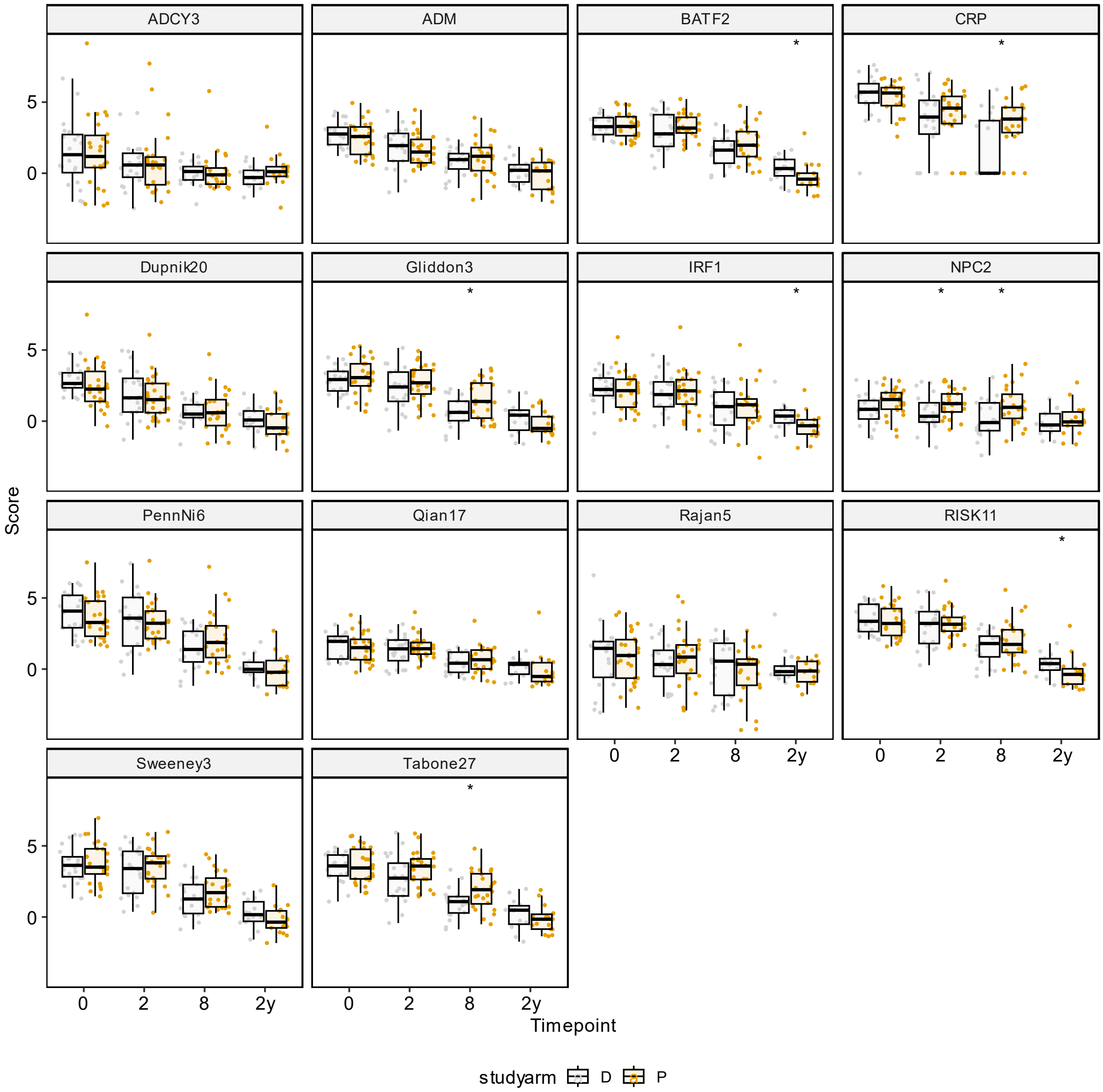

Blood RNA signature scores stratified by time and AdjuVIT study arm

Blood RNA signature Z scores are shown stratified by time point (0-8 weeks and 2 years follow up) and AdjuVIT trial intervention arm to receive vitamin D or placebo.

Supplementary Figure 5

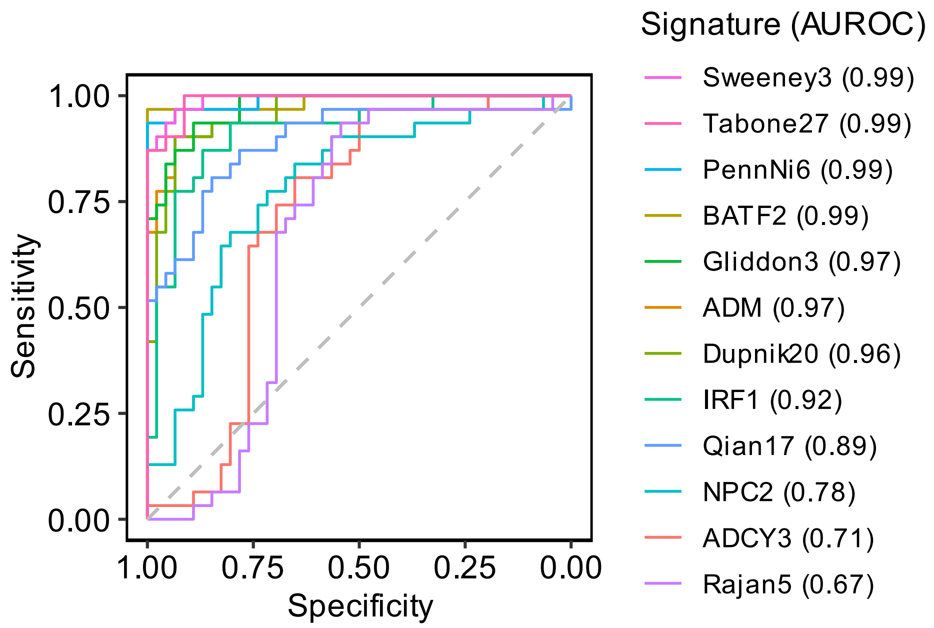

Blood RNA signature discrimination of pre-treatment and 2-year post treatment samples from AdjuVIT study.

Receiver operating characteristic curves for discrimination of pre-treatment samples and 2-year post-treatment samples from the AdjuVITs tidy by blood RNA signatures shown (with AUROC point estimates).

Supplementary Table 3

Signature	Reported AUROC in original publication	Observed AUROC in present data	Youden index		Z > 2	
			Sensitivity	Specificity	Sensitivity	Specificity
Sweeney3	0.90 (0.85-0.95)	0.99 (0.98-1.00)	0.97 (0.84-1.00)	0.93 (0.82-0.98)	0.91 (0.80-0.97)	0.97 (0.84-1.00)
Tabone27	0.96 (0.92-1.00)	0.99 (0.98-1.00)	1.00 (0.89-1.00)	0.91 (0.80-0.97)	0.91 (0.80-0.97)	1.00 (0.89-1.00)
PennNi6	0.94 (0.86-1.00)	0.99 (0.97-1.00)	0.94 (0.79-0.98)	1.00 (0.92-1.00)	0.85 (0.72-0.92)	0.97 (0.84-1.00)
BATF2*	0.99 (0.96-1.00)	0.99 (0.96-1.00)	0.97 (0.84-1.00)	1.00 (0.92-1.00)	0.98 (0.89-1.00)	0.97 (0.84-1.00)
Gliddon3	0.97 (0.93-1.00)	0.97 (0.94-1.00)	0.94 (0.79-0.98)	0.89 (0.77-0.95)	0.78 (0.64-0.88)	0.94 (0.79-0.98)
ADM	0.90	0.97 (0.94-1.00)	0.90 (0.75-0.97)	0.93 (0.82-0.98)	0.72 (0.57-0.83)	1.00 (0.89-1.00)
Dupnik20	NA	0.96 (0.92-1.00)	0.90 (0.75-0.97)	0.93 (0.82-0.98)	0.70 (0.55-0.81)	0.97 (0.84-1.00)
IRF1	0.80	0.92 (0.85-0.98)	0.87 (0.71-0.95)	0.87 (0.74-0.94)	0.61 (0.46-0.74)	0.94 (0.79-0.98)
Qian17	0.99 (0.98-1.00)	0.89 (0.81-0.97)	0.81 (0.64-0.91)	0.85 (0.72-0.92)	0.35 (0.23-0.49)	0.97 (0.84-1.00)
NPC2	0.94 (0.81-1.00)	0.78 (0.67-0.89)	0.77 (0.60-0.89)	0.72 (0.57-0.83)	0.20 (0.11-0.33)	0.97 (0.84-1.00)
ADCY3	0.88 (0.83-0.93)	0.71 (0.59-0.83)	0.97 (0.84-1.00)	0.50 (0.36-0.64)	0.41 (0.28-0.56)	0.97 (0.84-1.00)
Rajan5	0.87 (0.72-0.98)	0.67 (0.54-0.80)	0.94 (0.79-0.98)	0.54 (0.40-0.68)	0.24 (0.14-0.38)	0.97 (0.84-1.00)

Performance metrics of blood RNA signatures for discrimination of pre-treatment and 2-year post treatment samples from AdjuVIT study.

Reported AUROC to discriminate active TB from healthy controls for individual blood RNA signatures are compared to observed AUROC for blood RNA signatures discrimination of pre-treatment and 2-year post treatment samples from AdjuVIT study. Sensitivity and specificity metrics are shown for the Youden Index of the AUROC curve, and using blood RNA signature Z score threshold of >2 (standardised against the distribution of blood RNA signature scores from the 2-year post treatment samples). Point estimates and 95% confidence intervals are shown for each metric.

….

Supplementary Figure 6

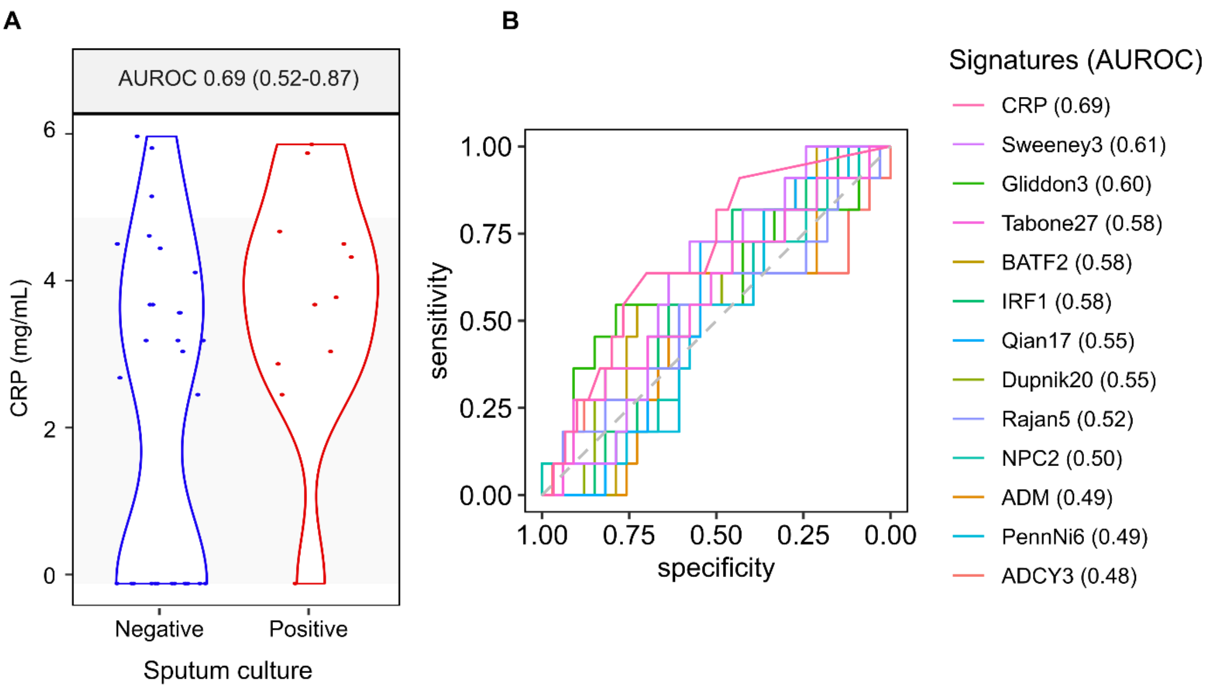

Biomarker discrimination of contemporary sputum culture result at 8 weeks TB treatment.

(A) Blood C-reactive protein (CRP) measurements stratified by contemporary sputum culture result at 8 weeks of treatment (showing AUROC point estimate and 95% confidence interval for between group discrimination). **(B)** Receiver operating characteristic curve analysis for blood RNA signatures and CRP discrimination of contemporary sputum culture positive and negative cases at 8 weeks TB treatment, showing AUROC point estimates for each biomarker.

Supplementary Table 4

		Max Youden Index		Z > 2	
**Signature**	**AUROC**	**Sensitivity**	**Specificity**	**Sensitivity**	**Specificity**
Sweeney3	0.61 (0.44-0.79)	0.73 (0.43-0.90)	0.58 (0.41-0.73)	0.73 (0.43-0.90)	0.45 (0.30-0.62)
Gliddon3	0.60 (0.38-0.82)	0.55 (0.28-0.79)	0.79 (0.62-0.89)	0.82 (0.52-0.95)	0.30 (0.17-0.47)
Tabone27	0.58 (0.38-0.79)	0.36 (0.15-0.65)	0.82 (0.66-0.91)	0.73 (0.43-0.90)	0.36 (0.22-0.53)
BATF2	0.58 (0.40-0.76)	0.55 (0.28-0.79)	0.73 (0.56-0.85)	0.73 (0.43-0.90)	0.42 (0.27-0.59)
IRF1	0.58 (0.40-0.76)	0.73 (0.43-0.90)	0.55 (0.38-0.70)	0.82 (0.52-0.95)	0.24 (0.13-0.41)
Qian17	0.55 (0.37-0.73)	0.73 (0.43-0.90)	0.55 (0.38-0.70)	1.00 (0.74-1.00)	0.03 (0.00-0.15)
Dupnik20	0.55 (0.35-0.74)	0.36 (0.15-0.65)	0.82 (0.66-0.91)	0.00 (0.00-0.26)	0.88 (0.73-0.95)
Rajan5	0.52 (0.31-0.73)	0.64 (0.35-0.85)	0.52 (0.35-0.67)	0.18 (0.05-0.48)	0.91 (0.76-0.97)
NPC2	0.50 (0.30-0.69)	0.73 (0.43-0.90)	0.39 (0.25-0.56)	0.91 (0.62-1.00)	0.18 (0.09-0.34)
ADM	0.49 (0.30-0.68)	1.00 (0.74-1.00)	0.18 (0.09-0.34)	1.00 (0.74-1.00)	0.18 (0.09-0.34)
PennNi6	0.49 (0.31-0.67)	0.82 (0.52-0.95)	0.36 (0.22-0.53)	0.45 (0.21-0.72)	0.58 (0.41-0.73)
ADCY3	0.48 (0.26-0.71)	0.27 (0.10-0.57)	0.88 (0.73-0.95)	0.00 (0.00-0.26)	0.97 (0.85-1.00)
CRP	0.69 (0.52-0.87)	0.91 (0.62-1.00)	0.43 (0.27-0.61)	0.91 (0.62-1.00)	0.43 (0.27-0.61)

Performance metrics of blood RNA signatures for discrimination of sputum culture positive and negative cases at 8 weeks TB treatment.

Observed AUROC for blood RNA signatures discrimination of sputum culture positive and negative cases at 8 weeks TB treatment from AdjuVIT study. Sensitivity and specificity metrics are shown for the Youden Index of the AUROC curve, and using blood RNA signature Z score threshold of >2 (standardised against the distribution of blood RNA signature scores from the 2-year post treatment samples). Point estimates and 95% confidence intervals are shown for each metric.

Supplementary Figure 7

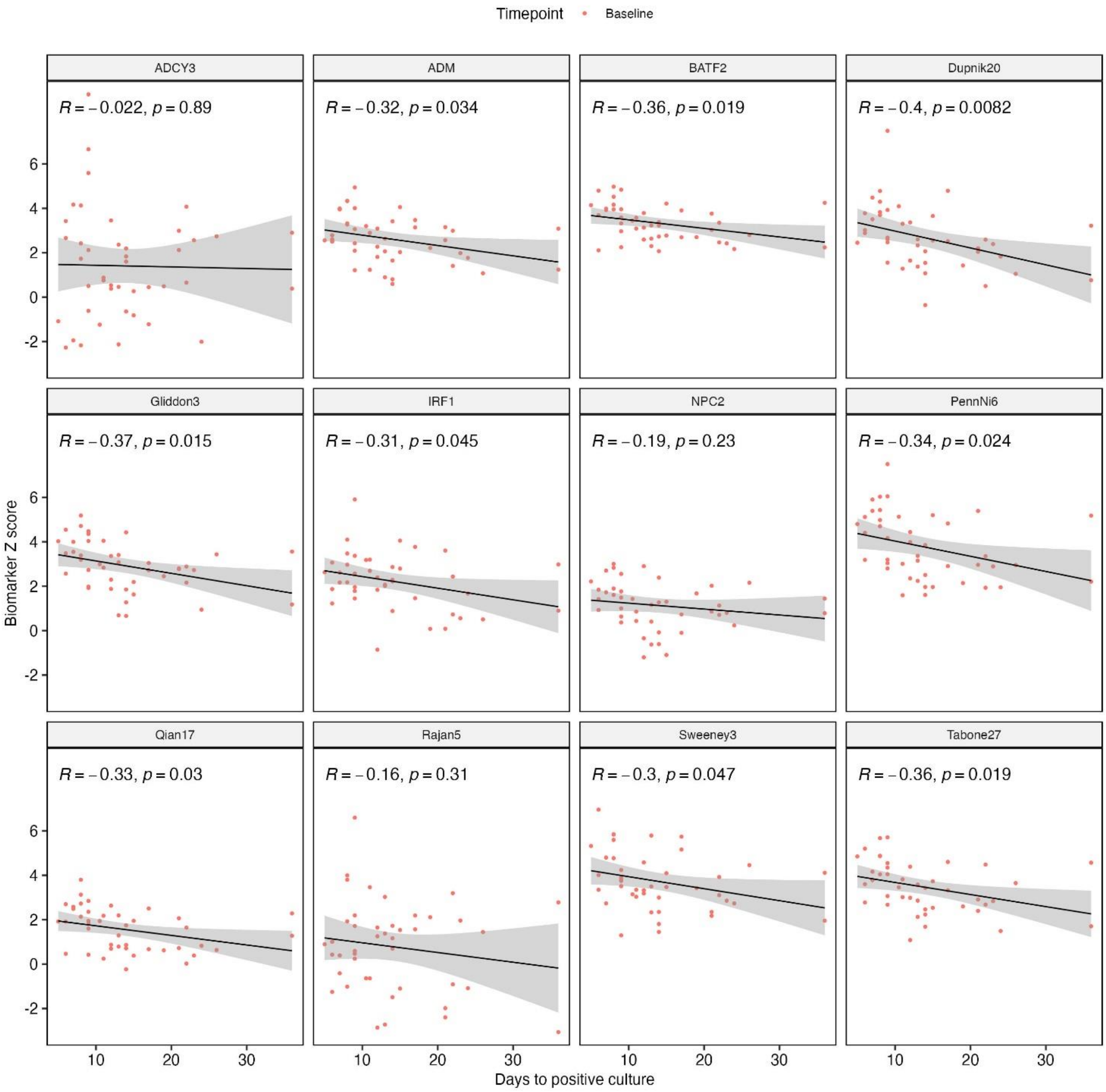

<u>Correlation between pre-treatment blood RNA signature scores and sputum culture time to positivity</u>

Scatter plots showing blood RNA signature Z scores and sputum culture time to positivity are shown with linear regression lines (±95% confidence inervals) with Spearman R correlation coefficient p-values.

Supplementary Figure 8

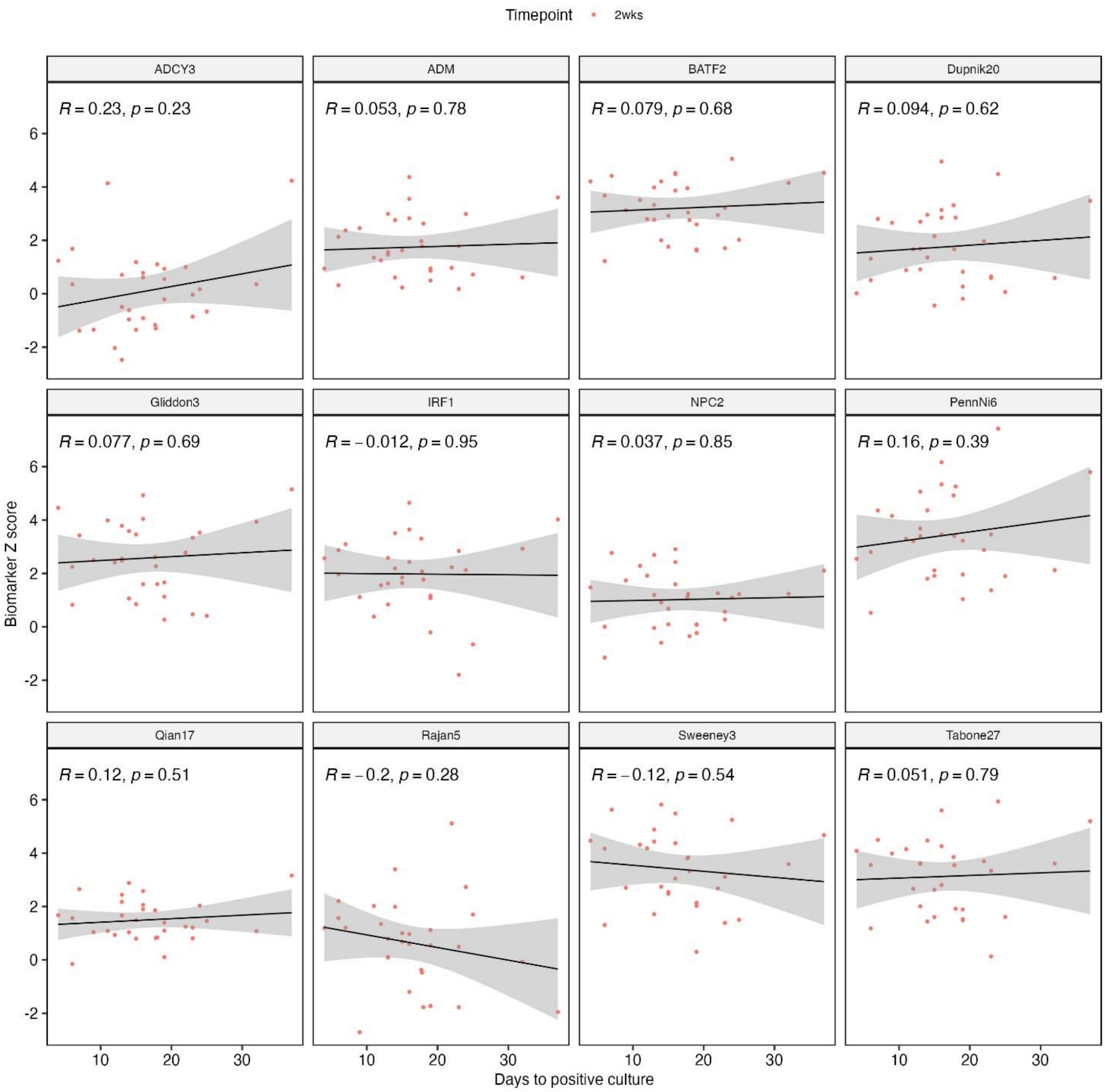

Correlation between blood RNA signature scores and sputum culture time to positivity at 2 weeks after treatment initiation.

Scatter plots showing blood RNA signature Z scores and sputum culture time to positivity among culture positive participants at 2 weeks after treatment initiation are shown with linear regression lines (±95% confidence inervals) with Spearman R correlation coefficient p-values

Supplementary Figure 9

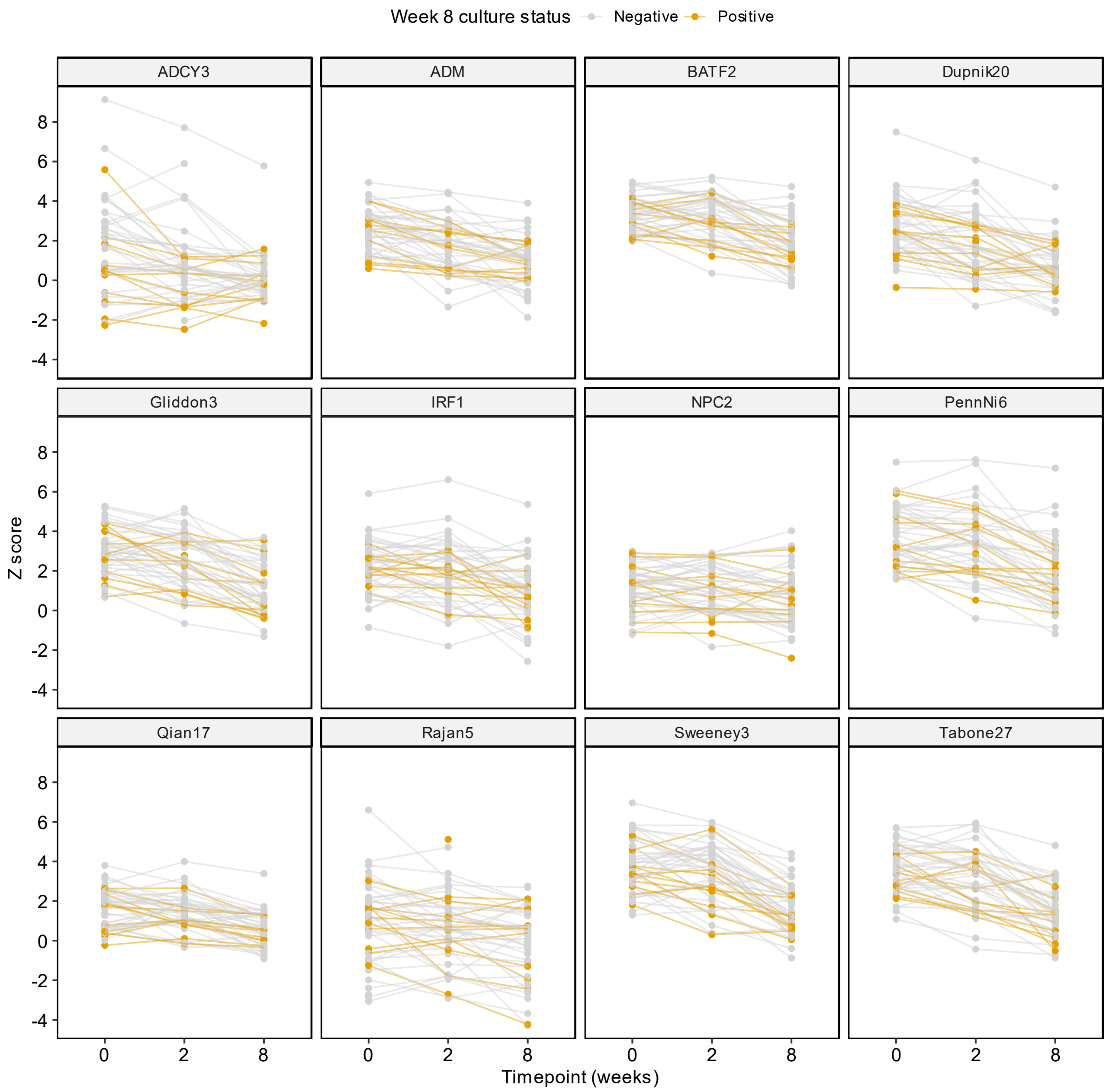

Longitudinal blood RNA signature scores stratified by sputum culture status at 8 weeks TB treatment.

Longitudinal blood RNA signature Z scores (standardised against the distribution of blood RNA signature scores from the 2-year post treatment samples) are shown pre-treatment and 2 and 8 weeks TB treatment at for individual participants stratified by sputum culture status 8 weeks of TB treatment.

Supplementary Figure 10

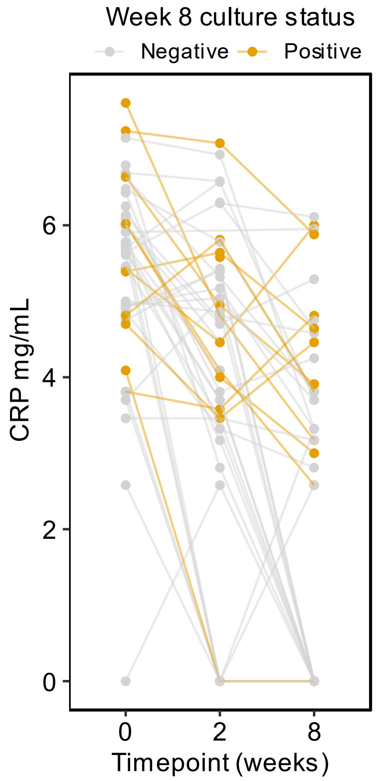

Longitudinal CRP measurements stratified by sputum culture status at 8 weeks TB treatment.

Longitudinal C-reactive protein measurements are shown pre-treatment and 2 and 8 weeks TB treatment at for individual participants stratified by sputum culture status 8 weeks of TB treatment.

Supplementary Table 5

**Signature**	**AUROC (Baseline)**	**AUROC (2 weeks)**	**AUROC (8wks - Baseline)***
ADCY3	0.66 (0.48-0.85)	0.72 (0.55-0.89)	0.65 (0.45-0.85)
ADM	0.61 (0.39-0.83)	0.61 (0.43-0.79)	0.64 (0.45-0.83)
BATF2	0.45 (0.25-0.65)	0.65 (0.47-0.84)	0.53 (0.35-0.72)
CRP	0.46 (0.24-0.68)	0.59 (0.40-0.77)	0.68 (0.50-0.86)
Dupnik20	0.56 (0.35-0.77)	0.47 (0.29-0.65)	0.65 (0.46-0.84)
Gliddon3	0.56 (0.34-0.77)	0.62 (0.44-0.80)	0.51 (0.28-0.73)
IRF1	0.53 (0.36-0.70)	0.55 (0.38-0.72)	0.56 (0.38-0.74)
NPC2	0.46 (0.23-0.69)	0.55 (0.35-0.76)	0.50 (0.32-0.68)
PennNi6	0.57 (0.35-0.79)	0.58 (0.38-0.78)	0.44 (0.23-0.64)
Qian17	0.40 (0.19-0.62)	0.71 (0.54-0.88)	0.55 (0.36-0.74)
RISK11	0.59 (0.39-0.79)	0.65 (0.48-0.82)	0.54 (0.35-0.73)
Rajan5	0.51 (0.33-0.68)	0.52 (0.32-0.71)	0.51 (0.28-0.73)
Sweeney3	0.60 (0.42-0.78)	0.71 (0.53-0.88)	0.55 (0.38-0.73)
Tabone27	0.60 (0.42-0.78)	0.63 (0.46-0.81)	0.48 (0.28-0.68)

Discrimination of sputum culture status at 8 weeks by blood RNA measurements at baseline, 2 weeks post treatment initiation and change in levels between baseline and 8 week post treatment initiation.

Table showing AUROC for biomarker measured at timepoint indicated, to predict 8-week culture conversion. Column indicated `8wks – Baseline` is assessing the diagnostic accuracy of the absolute difference in biomarker scores between baseline and 8 weeks for 8 week culture conversion.

Supplementary Figure 11

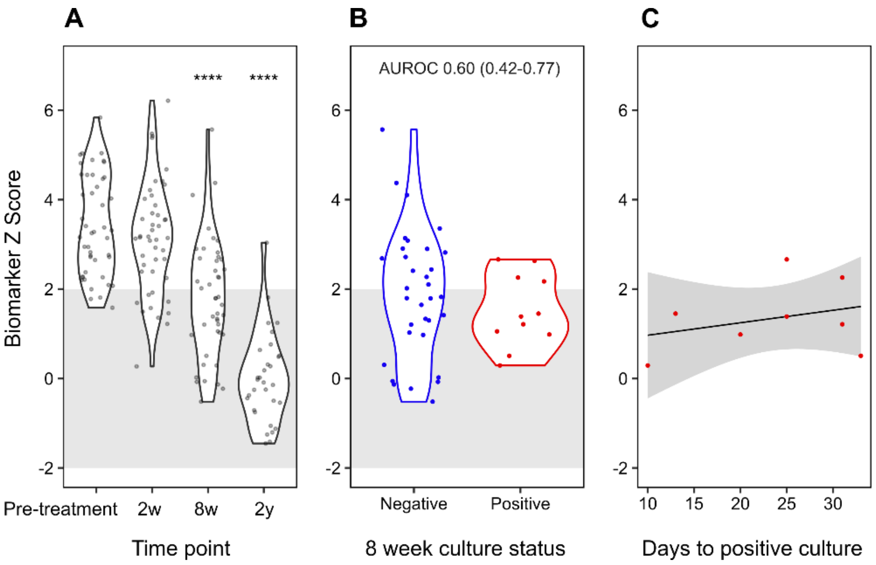

__Performance of modified Darboe11 signature__

(A) Signature scores standardised to the distribution of 2 year post-treatment samples are shown pre-treatment, and 2 weeks, 8 weeks and 2 years (2y) from initiation of treatment. 2 week, 8 week and 2 year time points were compared to pre-treatment by Wilcoxon Rank Sum test. *p values for change from baseline to timepoint indicated with * p <0.05, ** p <0.01 *** p <0.001 and **** p <0.0001. Grey shaded area indicates Z-score +/-2 (normalised to 2-year values). **(B)** Distributions of signature scores at 8 weeks of treatment for tuberculosis, stratified by contemporary sputum culture status, showing AUROC and associated 95% confidence intervals) for each signature. **(C)** Scatter plot of signature scores with sputum culture time (days) to positivity as a surrogate of bacterial load at 8 weeks of TB treatment showing individual data points and linear model (with 95% confidence intervals) for sputum culture positive cases.
