# Supplementary material for: Resolution of tuberculosis blood RNA signatures fails to discriminate persistent sputum culture positivity after 8 weeks of anti-tuberculous treatment
Source: Eur Respir J. 2024 Nov 21;64(5):2400457. doi: 10.1183/13993003.00457-2024 (PMC11579542; doi:10.1183/13993003.00457-2024)
Supplement: Supplementary file 3 [file ERJ-00457-2024.Shareable.pdf]

SHAREABLE PDF

Resolution of tuberculosis blood RNA signatures fails to discriminate persistent sputum culture positivity after 8 weeks of anti-tuberculous treatment

Claire J. Calderwood, Alvaro Sanchez Martinez, James Greenan-Barrett, Carolin T. Turner, Blanché Oguti, Jennifer K. Roe, Rishi Gupta, Adrian R. Martineau and Mahdad Noursadeghi

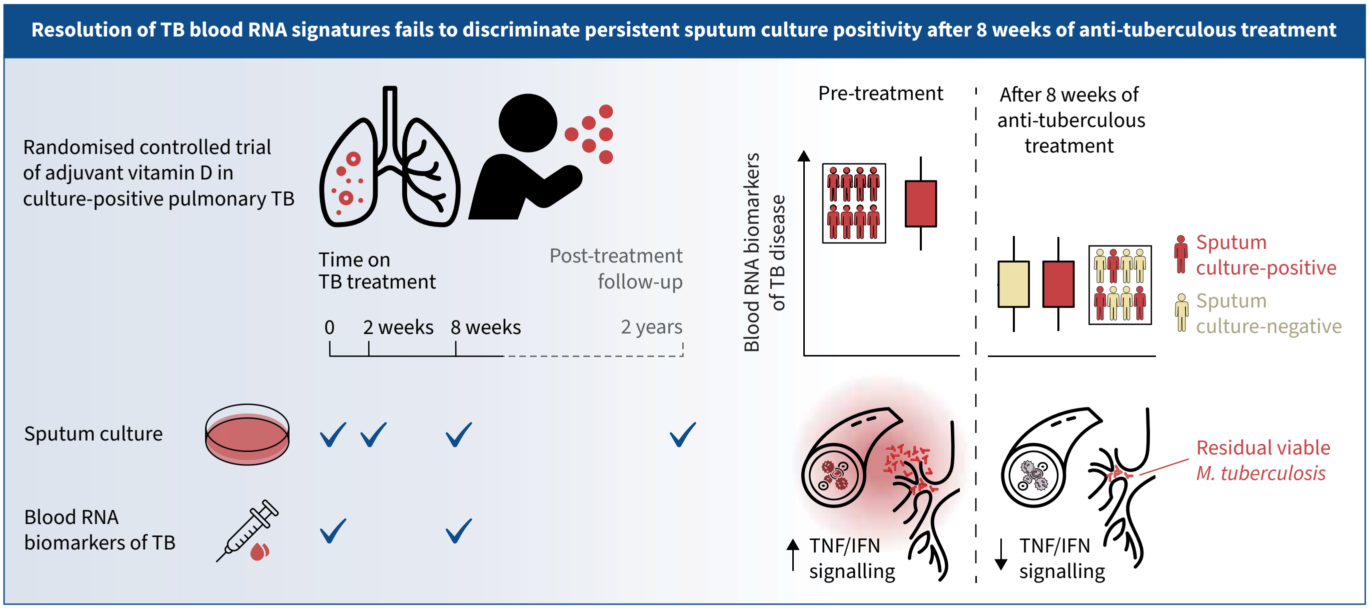

GRAPHICAL ABSTRACT Overview of the study. TB: tuberculosis; TNF: tumour necrosis factor; IFN: interferon; *M. tuberculosis*: *Mycobacterium tuberculosis*.

EUROPEAN RESPIRATORY JOURNAL
ORIGINAL RESEARCH ARTICLE
C.J. CALDERWOOD ET AL.

SHAREABLE PDF

Resolution of tuberculosis blood RNA signatures fails to discriminate persistent sputum culture positivity after 8 weeks of anti-tuberculous treatment

Claire J. Calderwood[1,6], Alvaro Sanchez Martinez[2,6], James Greenan-Barrett[3], Carolin T. Turner[2], Blanché Oguti[2], Jennifer K. Roe[2], Rishi Gupta[4], Adrian R. Martineau[5,7] and Mahdad Noursadeghi[2,7]

[1]Faculty of Infectious and Tropical Diseases, London School of Hygiene and Tropical Medicine, London, UK. [2]Division of Infection and Immunity, University College London, London, UK. [3]Department of Respiratory Medicine, University College London Hospitals NHS Foundation Trust, London, UK. [4]Institute of Health Informatics, University College London, London, UK. [5]Blizard Institute, Queen Mary University of London, London, UK. [6]C.J. Calderwood and A. Sanchez Martinez contributed equally. [7]A.R. Martineau and M. Noursadeghi are co-senior authors.

Corresponding author: Adrian R. Martineau (a.martineau@qmul.ac.uk)

Shareable abstract (@ERSpublications)
Resolution of current blood RNA biomarkers of tuberculosis to normal levels is insufficient to stratify patients for treatment cessation at 8 weeks, and illustrates uncoupling of host responses from persistence of viable bacteria in the respiratory tract https://bit.ly/4bN2b1U

Cite this article as: Calderwood CJ, Sanchez Martinez A, Greenan-Barrett J, *et al*. Resolution of tuberculosis blood RNA signatures fails to discriminate persistent sputum culture positivity after 8 weeks of anti-tuberculous treatment. *Eur Respir J* 2024; 64: 2400457 [DOI: 10.1183/13993003.00457-2024].

This extracted version can be shared freely online.

Copyright ©The authors 2024.

This version is distributed under the terms of the Creative Commons Attribution Licence 4.0.

This article has an editorial commentary: https://doi.org/10.1183/13993003.01669-2024

Received: 5 March 2024
Accepted: 9 July 2024

Abstract

Background Concerted efforts aim to reduce the burden of 6 months of anti-tuberculous treatment for tuberculosis (TB). Treatment cessation at 8 weeks is effective for most but incurs increased risk of disease relapse. We tested the hypothesis that blood RNA signatures or C-reactive protein (CRP) measurements discriminate 8-week sputum culture status, as a prerequisite for a biomarker to stratify risk of relapse following treatment cessation at this time-point.

Methods We identified blood RNA signatures of TB disease or cure by systematic review. We evaluated these signatures and CRP measurements in a pulmonary TB cohort, pre-treatment, at 2 and 8 weeks of treatment, and sustained cure after treatment completion. We tested biomarker discrimination of 8-week sputum culture status using area under the receiver operating characteristic curve (AUROC) analysis and, secondarily, assessed correlation of biomarker scores with time to culture positivity at 8 weeks of treatment.

Results 12 blood RNA signatures were reproduced in the dataset from 44 individuals with sputum culture-positive pulmonary TB. These normalised over time from TB treatment initiation. 11 out of 44 cases with blood RNA, CRP and sputum culture results were sputum culture-positive at 8 weeks of treatment. None of the contemporary blood RNA signatures discriminated sputum culture status at this time-point or correlated with bacterial load. CRP achieved modest discrimination with AUROC 0.69 (95% CI 0.52–0.87).

Conclusions Selected TB blood RNA signatures and CRP do not provide biomarkers of microbiological clearance to support TB treatment cessation at 8 weeks. Resolution of blood transcriptional host responses in sputum culture-positive individuals suggests *Mycobacterium tuberculosis* may colonise the respiratory tract without triggering a detectable immune response.
